# The mechanism and therapeutic potential of SIRT3 in central nervous system diseases: a review

**DOI:** 10.3389/fphar.2025.1652296

**Published:** 2025-09-03

**Authors:** Chuanbin Hong, Yupeng Wei, Yuchen Wang, Guangfu Lv, Xinglu Dong, Xiaowei Huang

**Affiliations:** ^1^ School of Pharmacy, Changchun University of Chinese Medicine, Changchun, China; ^2^ Department of Neurology, Dongzhimen Hospital, Beijing University of Chinese Medicine, Beijing, China; ^3^ Beijing University of Chinese Medicine, Beijing, China; ^4^ Pharmacology Group of Traditional Chinese Medicine, Jilin Ginseng Research Academy, Changchun University of Chinese Medicine, Changchun, China; ^5^ Affiliated Hospital of Changchun University of Chinese Medicine, Changchun, China; ^6^ Northeast Asia Research Institute of Traditional Chinese Medicine, Changchun University of Chinese Medicine, Changchun, China

**Keywords:** sirt3, silent information regulator 3, sirtuin-3, central nervous system diseases, mitochondrial function, therapeutic potential

## Abstract

Sirtuin-3 (SIRT3) is a mitochondrial deacetylase highly expressed in the nervous system, known to regulate mitochondrial homeostasis, energy metabolism, neuroinflammation, apoptosis, and oxidative stress, suggesting its potential neuroprotective role in central nervous system (CNS) disorders. Recent studies indicated that SIRT3 improves neuronal survival by reducing oxidative damage, alleviating neuroinflammation, and modulating autophagy. Therefore, it is imperative to conduct more in-depth and extensive investigations into the mechanisms underlying SIRT3 in central nervous system disorders. This review summarized current research advances on SIRT3, including its fundamental molecular structure, key downstream targets, and mechanisms of action in certain CNS diseases. It further analyzed the potential pharmacological mechanisms of several SIRT3 agonists and explored their therapeutic value in improving CNS disorders. Based on existing evidence, SIRT3 emerges as a promising therapeutic target, offering novel strategies for treating neurological diseases.

## 1 Introduction

In recent years, research on the pathological mechanisms of central nervous system (CNS) diseases has increasingly focused on the regulatory network of mitochondrial dysfunction and oxidative stress imbalance (Rakshe et al., 2024; Lautrup et al., 2019). The mitochondrial deacetylase Sirtuin-3 (SIRT3) has been identified as a critical regulator of metabolic systems, demonstrating its essential function in sustaining mitochondrial efficiency and coordinating cellular bioenergetics (Trinh et al., 2024). SIRT3 governs key physiological processes including mitochondrial quality control, oxidative stress resistance, energy metabolism, and apoptosis by mediating protein deacetylation (Meng et al., 2019). By activating superoxide dismutase 2 (SOD2), SIRT3 reduces reactive oxygen species (ROS) accumulation, thereby suppressing neuroinflammation and oxidative damage to enhance neuronal survival (Tyagi and Pugazhenthi, 2023). Furthermore, SIRT3 regulates mitophagy by activating downstream signaling pathways, including peroxisome proliferator-activated receptor γ coactivator-1α (PGC-1α), to promote mitochondrial biogenesis and maintain mitochondrial homeostasis (Li Y. et al., 2018). Additionally, SIRT3 modulates the NAD^+^/NADH ratio to enhance the tricarboxylic acid cycle and oxidative phosphorylation, ensuring neuronal energy supply and slowing disease progression (Cheng et al., 2021; Zhang et al., 2023). Notably, SIRT3 expression progressively decreases with advancing age, correlating with increased neurodegenerative disease susceptibility (Munteanu et al., 2024). Evidence from animal study demonstrated that exogenous upregulation of SIRT3 effectively ameliorated cognitive dysfunction in transgenic mouse models of Alzheimer’s disease (AD) (Yin et al., 2018a). These findings highlighted that SIRT3 emerged as a druggable target with significant clinical potential, while pharmacological activation of this mitochondrial deacetylase may represent a novel therapeutic avenue for treating CNS disorders. Therefore, further investigation of the upstream regulatory mechanisms of SIRT3 and its interactions with other cellular signaling pathways will enhance comprehension of the pathological mechanisms underlying CNS diseases while providing innovative conceptual frameworks for developing preventive and therapeutic strategies.

## 2 Physiological basis of SIRT3

### 2.1 Structure

SIRT3 is a mitochondrial NAD^+^-dependent deacetylase that plays critical roles in cellular energy metabolism, oxidative stress response, and apoptosis regulation (Shen et al., 2020). The gene of SIRT3 is located on human chromosome 11p15.5, encoding a 399-amino acid protein with a full-length molecular weight of approximately 44 kDa. Upon mitochondrial translocation, the mitochondrial targeting sequence (MTS) is cleaved, yielding mature SIRT3 with a molecular weight of about 28 kDa (Lescai et al., 2009; Donadini et al., 2013). The protein structure comprises a large domain and a small domain connected by a flexible loop region, forming a stable catalytic core. The large domain contains a Rossmann fold responsible for NAD^+^ binding and providing fundamental structural elements for catalytic activity, while the small domain stabilizes NAD^+^ binding and regulates substrate access to the catalytic pocket (Zhang J. et al., 2020). Additionally, the C-terminal region mediates protein-protein interactions to enhance substrate specificity or modulate enzymatic activity (Jin et al., 2009a). The intricate structural architecture of SIRT3 underpins its multifunctional roles.

### 2.2 Cellular localization and catalytic properties

SIRT3 primarily localizes to the mitochondrial matrix, and its sub-cell localization is tightly regulated by the MTS, transmembrane transport systems, post-translational modifications (PTMs), and intracellular signaling pathways (Zhang et al., 2015). The mRNA of SIRT3 is translated by free ribosomes in the cytoplasm, producing a precursor protein containing the MTS. This sequence, approximately 30 amino acids in length and enriched in arginine and lysine, confers mitochondrial affinity (Bao et al., 2010; Jin et al., 2009a). Besides, it can be recognized by the translocase of the outer mitochondrial membrane and translocase of the inner mitochondrial membrane complexes, mediating the transmembrane transport of SIRT3 (Zhang J. et al., 2020). Upon entering the mitochondrial matrix, the MTS of SIRT3 is cleaved by mitochondrial processing peptidase (MPP) at residues Arg31 and Ala32, forming a mature protein with intact catalytic activity (Ansari et al., 2017). The mature SIRT3 predominantly anchors to mitochondrial matrix protein complexes and stabilizes through interactions with mitochondrial matrix proteins such as heat shock protein 60 and heat shock protein 70 (Yang et al., 2011; Hu et al., 2022) (Figure 2b).

**FIGURE 1:**
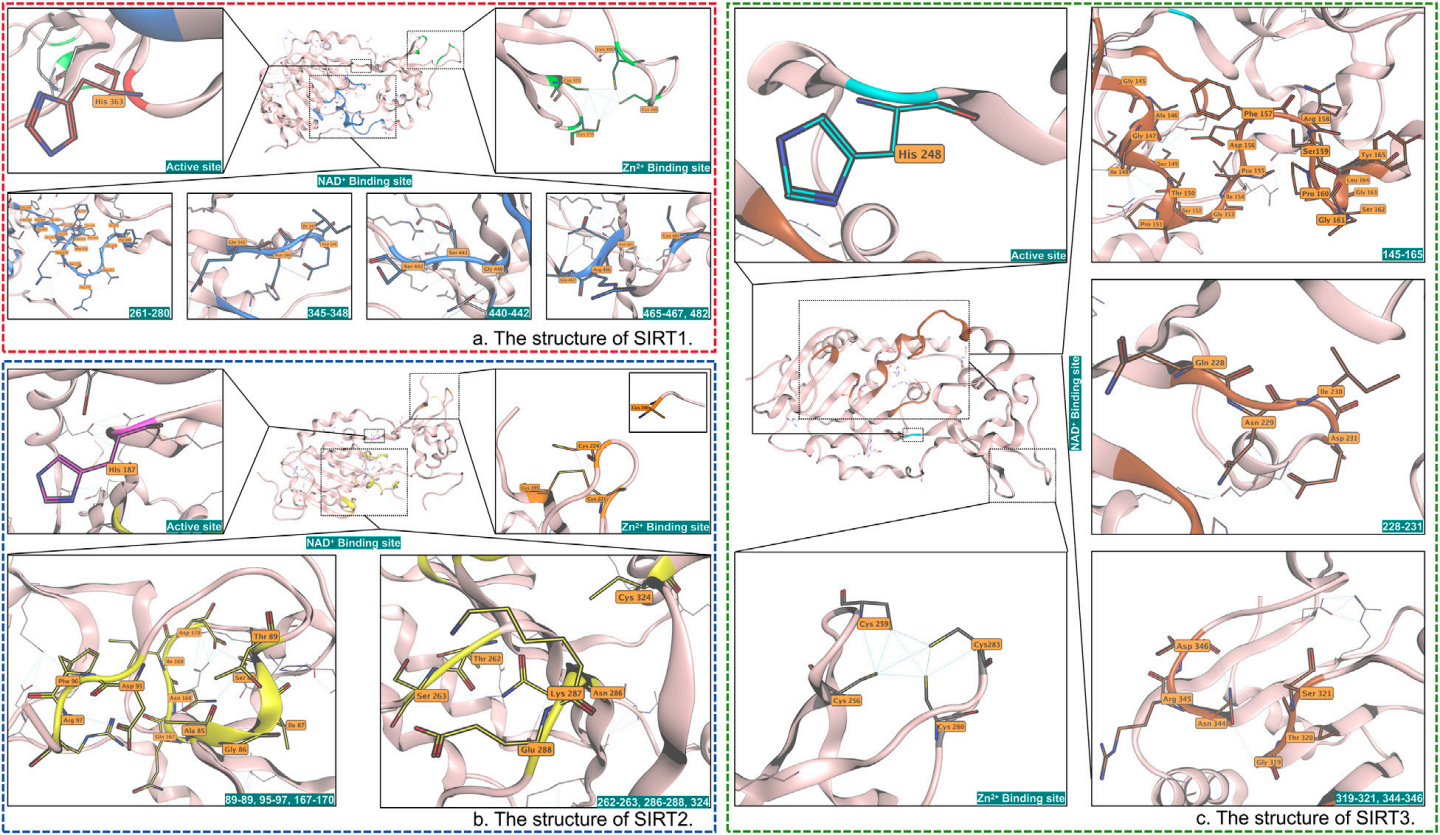
**(a)** The active site, Zn^2+^ binding site, and NAD^+^-binding site of SIRT1 are labeled red, green, and blue. **(b)** The active site, Zn^2+^ binding site, and NAD^+^ binding site of SIRT2 are labeled pink, orange, and yellow. **(c)** The active site, Zn^2+^ binding site, and NAD^+^ binding site of SIRT2 are labeled cyan, gray, and orange, respectively. SIRT3 specifically recognizes the β-hydroxy group and chiral center (preferring the S-configuration) of β-hydroxybutyrylation modification (Kbhb) through a hydrogen bond network in its active pocket, which is composed of residues H248, Q228, and V292. Meanwhile, the hydrophobic environment formed by F180, F294, I230, and V324 accommodates the acyl chain of Kbhb. Additionally, the hydrogen bond system consisting of E296, G295, and E325 forces the substrate peptide chain to adopt a β-sheet conformation, which, due to the entropic penalty effect, repels glycine flanking sites.

SIRT3 exhibited higher catalytic efficiency compared to Sirtuin-1 (SIRT1) and Sirtuin-2 (SIRT2). Firstly, mitochondria serve as the central hub for cellular energy metabolism and oxidative stress responses. Meanwhile, due to the hydrophobic pocket within the catalytic domain of SIRT3, SIRT3 can recognize complex groups (Zhang et al., 2019) (Figure 1), thereby enabling it to react with more substrates. Secondly, the catalytic activity of SIRT3 relies on NAD+ and involves multiple key amino acid residues, with His-248 serving as the core catalytic residue (Jin et al., 2009b). SIRT3 participates in physiological or pathological processes by mediating the deacetylation of diverse biological macromolecules (Lambona et al., 2024). Studies demonstrated that the enzymatic activity of SIRT3 is directly regulated by NAD + concentration and increasing proportionally with elevated NAD+/NADH ratios (Lambona et al., 2024; Feldman et al., 2015; Anderson et al., 2017).

### 2.3 Expression

**FIGURE 2:**
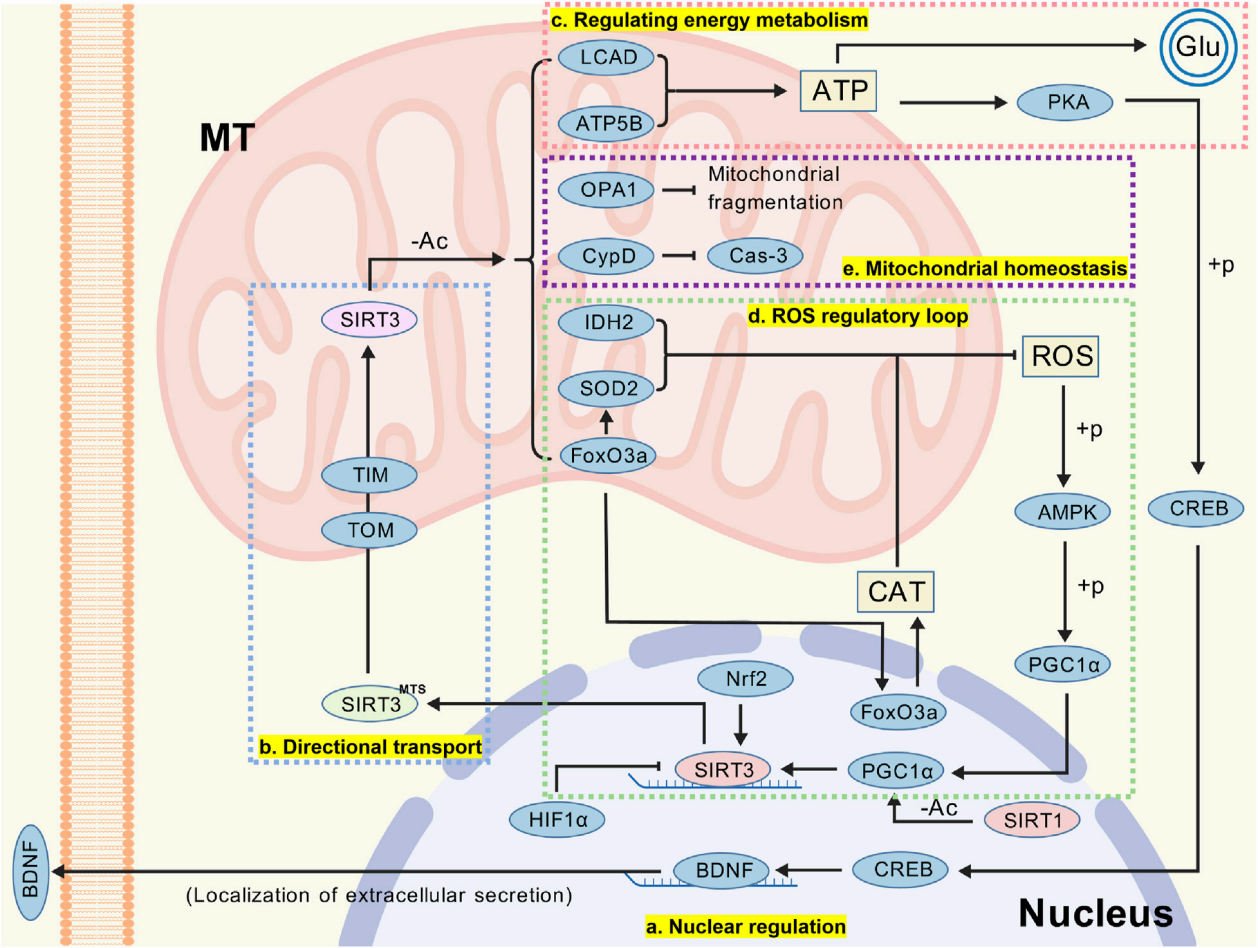
**(a)** SIRT3 expression and activity are regulated at multiple levels, including gene transcription, mRNA stability, post-translational modifications, protein stability, and enzyme activity regulation. **(b)** The MTS within the SIRT3 precursor is a critical fragment recognized and transported by TOM and TIM. After entering the mitochondria, the SIRT3 precursor undergoes cleavage to form mature SIRT3. **(c)** SIRT3 influences neuronal stability and information transmission by regulating ATP production. **(d)** SIRT3 plays a significant role in oxidative stress, and this article only illustrates one pathway through which SIRT3 modulates intracellular ROS homeostasis. **(e)** The cytoprotective effects of SIRT3 are also associated with its regulation of mitochondrial homeostasis. (Abbreviation: MT: mitochondria; SIRT3: Sirtuin-3; HIF-1α: hypoxia-inducible factor 1alpha; Nrf2: nuclear factor erythroid 2-related factor 2; MTS: mitochondrial targeting sequence; TOM: translocase of the outer mitochondrial membrane; TIM: translocase of the inner mitochondrial membrane; LCAD: long-chain acyl CoA dehydrogenase; ATP5B: ATP synthase β; Glu: glutamic acid; PKA: protein kinase A; CREB: cAMP-response element binding protein; BDNF: brain-derived neurotrophic factor; OPA1: optic atrophy 1; CypD: cyclophilin D; Cas-3: caspase-3; ROS: reactive oxygen species; SOD2: superoxide dismutase 2; IDH2: isocitrate dehydrogenase 2; FoxO3a: forkhead box O3; CAT: catalase; AMPK: adenosine monophosphate-activated protein kinase; PGC-1α: peroxisome proliferator-activated receptor γ coactivator 1 α).

The expressions of SIRT3 are regulated at multiple levels, including gene transcription, mRNA stability, PTMs, protein stability, and enzymatic activity. These mechanisms work synergistically under diverse physiological and pathological conditions to maintain mitochondrial metabolic homeostasis and cellular energy balance. For example, the promoter region of the SIRT3 gene contains multiple transcription factor binding sites. It is positively regulated by nuclear factor erythroid 2-related factor 2 (Nrf2), while hypoxia-inducible factor 1alpha (HIF-1α) suppress its transcription under hypoxic or stress conditions (Loboda et al., 2009; Yao et al., 2022) (Figure 2a). Metabolic regulatory signals also influence the transcriptional level of SIRT3. Chronic exposure to biochemical stress or mitochondrial metabolic abnormalities can accumulate ROS intracellularly, generating excessive superoxide, hydrogen peroxide, and hydroxyl radicals (Albano, 2006). These sequentially activate the phosphorylation of adenosine monophosphate-activated protein kinase (AMPK) and PGC-1α, thereby enhancing SIRT3 transcription (Wang Y. et al., 2025; Wang et al., 2022). On the other hand, nuclear-localized SIRT1 also participates in the positive regulation of SIRT3 expression by deacetylating PGC-1α in the nucleus (Zhang et al., 2021; Rodgers et al., 2005) (Figure 2d).

### 2.4 Function and related pathways

SIRT3 modulates energy metabolism, antioxidant responses, and mitochondrial homeostasis by deacetylating specific sites on key metabolic enzymes (Table 1). SIRT3 enhances the activity of long-chain acyl CoA dehydrogenase (LCAD) through deacetylation, accelerating fatty acid β-oxidation and promoting the generation of more acetyl-CoA. Via the tricarboxylic acid cycle, this process boosts ATP production (Hirschey et al., 2010). For glutamatergic neurons, this step helps enhance synaptic transmission efficiency. On the other hand, deacetylation of ATP synthase β by SIRT3 strengthens its catalytic activity, improving the ATP synthesis efficiency at the terminal of the mitochondrial electron transport chain and increasing the availability of ATP in the cytoplasm to maintain synaptic vesicle cycling and neuronal electrical activity (Zhang et al., 2016). The increased ATP production also activates the protein kinase A/cAMP-response element binding protein (PKA/CREB) pathway, leading to CREB phosphorylation and promoting the expression of neurotrophic factors such as brain-derived neurotrophic factor (BDNF), which can enhance neuronal survival and plasticity (Mo et al., 2024) (Figure 2c).

**TABLE 1:** Specific sites of key metabolic enzymes involved in deacetylation of SIRT3.

Protein substrate	Deacetylation site	Function	References
LCAD	K42	Promote fatty acid oxidation	Hirschey et al. (2010)
ATP5B	K485	Synthesize ATP	Zhang et al. (2016)
SOD2	K68, K122	Enhance antioxidant stress ability	Dikalova et al. (2017)
IDH2	K413	Promote NADPH generation	Zou et al. (2017)
FoxO3a	K271, K290	Regulate mitochondrial oxidative stress	Wang J. et al. (2025)
CypD	K166	Regulate mPTP activity	Yan et al. (2022)
OPA1	K926, K931	Regulate mitochondrial fusion	Samant et al. (2014)

Abbreviation: SIRT3: Sirtuin-3; LCAD: long-chain acyl CoA dehydrogenase; ATP5B: ATP, synthase β; SOD2: superoxide dismutase 2; IDH2: isocitrate dehydrogenase 2; FoxO3a: forkhead box O3; CypD: cyclophilin D; OPA1: optic atrophy 1; NADPH: nicotinamide adenine dinucleotide phosphate; mPTP: mitochondrial permeability transition pore.

As previously mentioned, elevated ROS in the cytoplasm can increase SIRT3 expression by activating the AMPK/PGC-1α pathway. In turn, SIRT3 deacetylates SOD2 and isocitrate dehydrogenase 2 (IDH2), significantly enhancing their enzymatic activity to inhibit ROS (Dikalova et al., 2017; Zou et al., 2017). On the other hand, SIRT3-mediated deacetylation of forkhead box O3 (FoxO3a) in mitochondria activates FoxO3a-dependent gene expression (Jacobs et al., 2008). This not only regulates SOD2 to inhibit ROS but also enhances the activity of catalase, achieving clearance of hydrogen peroxide and reducing oxidative stress. Overall, SIRT3 plays a critical role in maintaining intracellular ROS homeostasis (Figure 2d).

Optic atrophy 1 (OPA1) is a critical regulatory protein involved in maintaining mitochondrial inner membrane fusion and the formation of mitochondrial cristae structures (von der Malsburg et al., 2023). The regulation of OPA1 by SIRT3 facilitates the repair and functional recovery of mitochondrial structures, preventing mitochondrial fragmentation (Chen et al., 2024b; Samant et al., 2014). The opening of the mitochondrial permeability transition pore (mPTP) is a hallmark of mitochondrial dysfunction, which is closely associated with excessive acetylation of cyclophilin D (CypD) (Dikalova et al., 2024). By deacetylating CypD, SIRT3 modulates mPTP activity to inhibit the release of cytochrome C, which suppresses the activation of Caspase-3 and ultimately inhibiting apoptosis (Yan et al., 2022; Poppe et al., 2001) (Figure 2e).

## 3 Role of SIRT3 in central neurons

**FIGURE 3:**
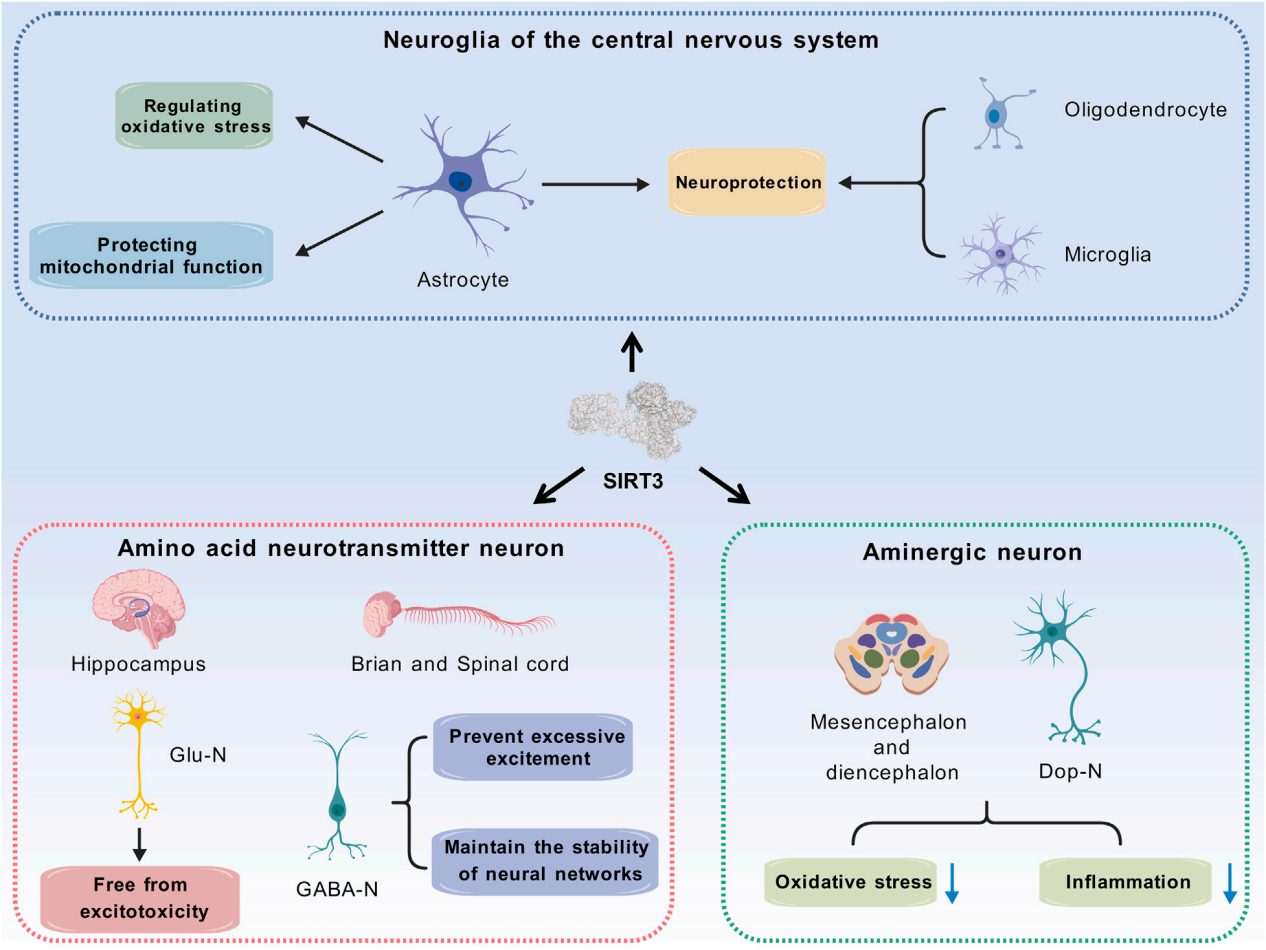
SIRT3 exerts distinct physiological functions in different types of cells. Glutamatergic neurons and GABAergic neurons serve as representative excitatory and inhibitory neurons, and the roles of SIRT3 in these 2 cell types contribute to maintaining excitatory-inhibitory homeostasis. Mitochondrial damage induced by reduced SIRT3 expression accelerates the degeneration of dopaminergic neurons, which may serve as a potential pathogenesis of Parkinson’s disease. As for neuroglia in the central nervous system, SIRT3 achieves neuroprotective effects through intracellular regulatory mechanisms. (Abbreviation: SIRT3: Sirtuin-3; Glu-N: Glutamatergic Neurons; GABA-N: GABAergic Neurons; Dop-N: Dopaminergic Neurons).

SIRT3 is widely distributed across various types of central neurons, and its expression and function are cell specific. Current research primarily focuses on glutamatergic neurons, GABAergic neurons, dopaminergic neurons, and astrocytes (Figure 3).

### 3.1 Glutamatergic neurons

Glutamate is the main excitatory neurotransmitter in the vertebrate brain. Its excessive release triggers membrane depolarization through receptor-mediated Na^+^ and Ca^2+^ influx, increasing neuronal mitochondrial oxidative phosphorylation and superoxide production (Rueda et al., 2016). A study on cortical mitochondria of SIRT3 knockout (KO) mice showed that the lack of SIRT3 affected the subcellular regulation of Ca^2+^ after glutamate induced Ca^2+^ influx and confirmed through neuronal cell and mouse running experiments that glutamatergic signaling mediates the upregulation of SIRT3. The bidirectional regulatory effect between SIRT3 and glutamatergic neurons enhanced the resistance to degeneration in hippocampal and cortical neurons (Cheng et al., 2016). The reduced incorporation of [1,6–13C]glucose-derived carbon into all isotopomers of glutamate, glutamine, γ-aminobutyric acid (GABA), and aspartate in SIRT3 knockout brains demonstrated diminished mitochondrial metabolic activity and tricarboxylic acid cycle flux in both neuronal and astrocytic compartments (Kristian et al., 2021). Continuous high levels of Ca^2+^ activated protein phosphatase 4, leading to high doses of glutamate inhibiting the AMPK/PGC-1α/SIRT3 pathway, while brief increased in Ca^2+^ levels could activate this pathway, providing important insights for precise regulation of glutamate and SIRT3 levels to alleviate brain related diseases (Gu et al., 2023). There are gender differences between glutamate and SIRT3. It was reported that the lack of SIRT3 significantly increased the expression of N-methyl-D-aspartate (NMDA) receptor in the hippocampus of female SIRT3 KO mice only. Excessive upregulation of NMDA receptor 2B could enhance glutamatergic excitotoxicity, affecting primary neural development and synaptic plasticity (Allen et al., 2023). Based on the complex relationship between glutamate and SIRT3, further exploration of strategies to balance their expression may provide novel therapeutic directions for neurological diseases.

### 3.2 GABAergic neurons

GABA is an inhibitory neurotransmitter that has effects on learning, sleep, memory, and muscle movement. Dysfunction and degeneration of GABAergic neurons lead to abnormal hyperexcitability of neural circuits, also causing degeneration of glutamatergic neurons (Palop and Mucke, 2016). After treatment with diazepam, the activation of GABA receptors significantly reduced seizures induced by kainic acid in SIRT3^+/−^ AppPs1 mice. The experiment also showed that the loss of GABAergic neurons and the exacerbation of neural network overexcitation were caused by a decrease in SIRT3 (Cheng et al., 2020). Intermittent fasting improves multiple health indicators and can slow down the progression of diabetes, vascular disorders, and AD (Mattson et al., 2018). Experiment on SIRT3 KO mice demonstrated that SIRT3 was essential for enhancing GABAergic synaptic transmission adaptability and counteracted anxiety during intermittent fasting (Liu et al., 2019). In rats with middle cerebral artery occlusion, the neuroprotective effects of wogonoside in cerebral ischemia-reperfusion injury were mediated through regulating GABAergic amino acid metabolism, mitochondrial bioenergetics, and glutathione biosynthesis pathways, which collectively preserved redox equilibrium while suppressing oxidative damage through attenuation of reactive oxygen species overproduction (Xu et al., 2024). In summary, GABAergic neurons not only ameliorate neurological disorders through interactions with SIRT3 but also exhibit complex interplay with glutamatergic neurons.

### 3.3 Dopaminergic neurons

Midbrain dopaminergic neurons have been a focal point of intensive research. The nigrostriatal dopamine system is crucial for coordinating fine motor skills and maintaining movement balance. The degeneration of dopaminergic neurons in the substantia nigra is an important pathological mechanism of Parkinson’s disease (PD) (Arenas et al., 2015). It was reported that SIRT3 KO mice exhibited significantly elevated acetylation levels of manganese superoxide dismutase (MnSOD) on lysine 68 in substantia nigra pars compacta dopaminergic neurons compared to wild-type (WT) mice, alongside its mitochondrial MnSOD activity was significantly lower than that of WT mice. This proves that in mouse substantia nigra pars compacta dopaminergic neurons, SIRT3 deacetylates MnSOD on lysine 68 to increase its activity and reduce mitochondrial oxidative stress (Shi et al., 2017). *In vitro* experiments using MN9D cells revealed that SIRT3 promoted mitochondrial autophagy while inhibiting activation of the nucleotide-binding oligomerization domain-like receptor protein 3 (NLRP3) inflammasome in dopaminergic neurons (Jiang et al., 2022). Additionally, SIRT3 also mitigated oxidative stress-induced neurotoxicity in dopaminergic neurons (Lee et al., 2021). For instance, SIRT3 directly deacetylated SOD2 and adenosine triphosphate (ATP) synthase β in dopaminergic neurons to prevent cell death (Zhang et al., 2016). In the rat model of subarachnoid hemorrhage, dopamine-D2-agonists were shown to inhibit mitochondrial fission mediated by dynamin-related protein 1, via activating mitofusin 2 and optic atrophy 1. On the other hand, dopamine-D2-agonists regulated PGC-1α/SIRT3 pathway by restricting cytochrome C in mitochondria, which could improve mitochondrial dysfunction and exert neuroprotective effects (Rehman et al., 2025). To summarize, these findings highlighted the bidirectional regulatory interactions between SIRT3 and dopaminergic neurons, which collectively maintain neuronal homeostasis.

### 3.4 Astrocytes

Astrocytes are widely distributed in the mammalian brain, and involve in maintaining the neurovascular unit, facilitating synaptic network formation, regulating ionic balance, regulating synaptic neurotransmitter concentrations, and synthesizing bioactive molecules that influence neuronal activity (Khakh and Deneen, 2019). Cell experiments showed that hyperglycemic conditions significantly enhanced the susceptibility of SIRT3 to recurrent low glucose, inducing mitochondrial structural abnormalities in astrocytes. However, overexpression of SIRT3 demonstrated preservation of mitochondrial bioenergetics while decreasing oxidative damage biomarkers induced by recurrent low glucose. Meanwhile, SIRT3 inhibited the transformation of astrocytes into neuroinflammatory A1 like reactive phenotype (Gao R. et al., 2022). It was also reported that SIRT3 could modify astrocyte activation by regulating the Notch1/NF-κB pathway, alleviating inflammatory responses following status epilepticus (Zhu et al., 2024). As for ischemic stroke, SIRT3 exerted a protective effect by regulating the HIF-1α/VEGF pathway in astrocytes (Yang X. et al., 2021). Furthermore, SIRT3 prevents astrocyte A1 polarization and associated neurotoxicity under chronic hypoxia by inhibiting phosphorylation and nuclear translocation of the transcription factor signal transducer and activator of transcription 3 (STAT3) (Hu et al., 2023). Research indicated that caffeine improved astrocyte-mediated protein Tau (Tau) neurotoxicity via modulation of the EGR1/SIRT3 pathway (Gao et al., 2024). In total, the roles of SIRT3 in astrocytes are complex and critical, demonstrating its potential to mitigate astrocyte injury and protect CNS targets.

### 3.5 Other neuron types

Astrocytes, oligodendrocytes, and microglia are the main glial cells in CNS. The main functions of oligodendrocytes include wrapping around axons, forming myelin sheaths, assisting in the efficient transmission of biological electrical signals, and maintaining the normal function of neurons (Emery and Wood, 2024). Microglia, characterized by their multipolar morphology and plasticity, are immune effector cells in CNS and play critical roles in physiological processes (Colonna and Butovsky, 2017). Studies demonstrated enhanced expression of SIRT3 in astrocytes, oligodendrocytes, and microglia within the white matter of hypoxic newborn rats. Meanwhile, early hypoxia induced intense SIRT3 expression in microglia (Li X. et al., 2018). Quantitative PCR analyses across neuron subtypes revealed that SIRT3 exhibits the highest expression in primary neurons, followed by astrocytes, while oligodendrocytes and microglia show the lowest levels. PGC-1α altered the expression level of SIRT3 in neurons. Among different types of cells in PGC-1α KO mice, the expression level of SIRT3 was highest in astrocytes but lower than in WT mice (Buck et al., 2017).

## 4 SIRT3 in central nervous system diseases

### 4.1 Ischemic stroke

Ischemic stroke is primarily caused by cerebral blood flow interruption due to large-vessel atherosclerosis, small-vessel lacunar infarction, or cardioembolism (Sommer, 2017), which makes the brain starve of oxygen and glucose, leading to neuronal energy metabolism failure and secondary cell damage (Qin et al., 2022). Recent studies demonstrated that SIRT3 could repair mitochondrial ultrastructure and membrane composition, promote mitochondrial biogenesis, and alleviate mitochondrial dysfunction by upregulating the expression and activity of optic atrophy 1 (Chen et al., 2024b). Besides, SIRT3 also inhibited the expression of voltage-dependent anion channel 1 and adenine nucleotide translocase 1, preventing abnormal opening of the mitochondrial permeability transition pore and reducing mitochondrial apoptosis during ischemic injury (Yang Y. et al., 2021). Metabolomic analysis showed a significant increase in GABA and glutathione levels in the brain after wogonoside treatment, confirming that SIRT3 not only had ability to mitigate oxidative stress but also alleviated excitotoxicity caused by excessive glutamate release (Xu et al., 2024). Also, SIRT3 protects the integrity of the blood-brain barrier (BBB) in ischemic stroke mice by regulating the HIF-1α/VEGF pathway in astrocytes, reducing inflammatory responses and neuronal apoptosis (Yang X. et al., 2021). SIRT3 can also promote the migration of microglia in ischemic stroke by increasing the expression of CX3C chemokine receptor 1 (Cao et al., 2019). Furthermore, SIRT3 promoted PINK1/Parkin mediated mitochondrial autophagy, increased microvascular density and the expression of VEGF A, and reduced neuronal apoptosis in cerebral ischemia-reperfusion model rats (Wei et al., 2023). On the other hand, overexpression of SIRT1 can improve mitochondrial respiratory chain dysfunction by enhancing the deacetylation activity of SIRT3, reflecting the synergistic effect of the two in restoring mitochondrial structure and function (Chen et al., 2024c). However, the direct molecular interaction between SIRT1 and SIRT3 is not yet clear, and the synergistic effect lacks causal validation through gene knockout or dual intervention experiments. In general, intervention studies validated the therapeutic potential of SIRT3 for ischemic stroke (Table 2), but it is still necessary to explore its targeting ability and provide new directions for clinical treatment.

**TABLE 2:** SIRT3 activators that could improve ischemic stroke found in nearly a decade.

Activator	Mechanism	References
Silbene glycoside	Modulate SIRT3/AMPK pathway	Li et al. (2021)
Trilobatin	Modulate SIRT3/TLR4/Nrf2 pathway	Gao et al. (2020)
LanCL1	Modulate Akt-PGC-1α-SIRT3 pathway	Xie et al. (2018)
Icariside II	Modulate Nrf2/SIRT3 pathway	Feng et al. (2018)
Luteolin	Modulate SIRT3/AMPK/mTOR pathway	Liu et al. (2020)
Notoginseng Leaf Triterpenes	Modulate SIRT1/2/3-FoxO3a-MnSOD/PGC-1α pathway	Xie et al. (2020)
Genipin	Modulate UCP2-SIRT3 pathway	Zhao et al. (2019)
4′-O-methylbavachalcone	Modulate SIRT3-PARP-1 pathway	Chen et al. (2024d)
Honokiol	Modulate SIRT3/Drp1 pathway	Zheng et al. (2023)
AFPR	Modulate SIRT3/Pink1/Parkin pathway	Wei et al. (2023)

Abbreviation: SIRT3: Sirtuin-3; AMPK: Adenosine 5′-monophosphate (AMP)-activated protein kinase; TLR4: Toll-like receptor 4; Nrf2: nuclear factor erythroid 2-related factor 2; LanCL1: lanthionine synthetase C-like protein 1; Akt: protein kinase B; PGC-1α: proliferator-activated receptor γ coactivator-1α; mTOR: mammalian target of rapamycin; FoxO3a: forkhead box O3; MnSOD: superoxide dismutase; UCP2: uncoupling protein 2; PARP-1: Poly (ADP-ribose) polymerase-1; Drp1: dynamin-related protein 1; AFPR: active fraction of *Polyrhachis vicina* (Roger); PINK1: PTEN, induced putative kinase 1; Parkin: Parkin protein.

Diabetic cerebral ischemia-reperfusion injury (CIRI) refers to the secondary brain tissue damage caused by the restoration of cerebral blood flow after ischemic interruption in diabetic patients, leading to more severe neurological dysfunction (Zhou et al., 2025). Its main pathological mechanisms include enhanced inflammatory responses, exacerbated oxidative stress, and mitochondrial dysfunction (Przykaza, 2021). It was found that in diabetic CIRI rats, the levels of SIRT1/SIRT3 are significantly reduced, accompanied by decreased levels of mitochondria-regenerating proteins such as PGC-1α, nuclear respiratory factor 1 (NRF1), and transcription factor A (TFAM). This suggests that diabetes may hinder mitochondrial regeneration by inhibiting the SIRT1/SIRT3-PGC-1α-NRF1-TFAM signaling pathway, thereby exacerbating cerebral ischemia-reperfusion injury in rats (Xin et al., 2025). Although research on this topic is limited, existing data still confirms the critical protective role of SIRT3 in CIRI. For example, melatonin can alleviate CIRI in diabetic mice by activating the protein kinase B (Akt)/SIRT3/SOD2 pathway and improving mitochondrial damage. However, when SIRT3 upregulation is suppressed by 3-TYP, these protective effects are attenuated, confirming that SIRT3 plays a key role in diabetic cerebral ischemia-reperfusion injury (Liu L. et al., 2021). Further studies have demonstrated that rapamycin can maintain mitochondrial dynamic balance by regulating the SIRT3-dynamin-related protein 1 (DRP1)/OPA1 signaling pathway, thereby improving CIRI in diabetic rats (Hei et al., 2023). Given all that, exploring the synergistic interactions of SIRT3 and its differential expression in various neuron types will provide strong support for precise treatment of CIRI. Additionally, as a comorbidity of diabetes, clarifying the mechanism of SIRT3 in diabetes is of great significance for halting the further progression of neurological damage.

### 4.2 Dementia

#### 4.2.1 Vascular dementia

Vascular dementia (VD) is characterized by ischemic or hemorrhagic damage to brain tissue caused by cerebrovascular lesions, manifesting as multiple lesions, white matter lesions, neuronal loss, and disrupted neural networks (Kuang et al., 2021). In central neurons, SIRT3 alleviates neuroinflammation and mitochondrial dysfunction following ischemic hypoxic brain injury by inhibiting the activation of pro-inflammatory microglia (Yan et al., 2025). Gastrodin, an active component extracted from the root of *Gastrodia elata Bl.*, has the ability to ameliorate brain tissue injury through multiple pathways (Xiao et al., 2023). It was shown that gastrodin increased SIRT3 expression in VD model rats and deacetylated mitochondrial TFAM at K5, K7, and K8 site, reversing mitochondrial dysfunction, alleviating oxidative stress, and reducing aging (Chen et al., 2024e). Additionally, gastrodin enhanced ATP production, superoxide dismutase activity, and glutathione levels via SIRT3 regulation, further mitigating mitochondrial dysfunction in VD (Shi et al., 2024). As mentioned earlier, SIRT3 inhibited A1 polarization in astrocyte by regulating STAT3 to reduce oxidative stress and subsequent synaptic damage (Hu et al., 2023). Autophagy plays an important role in the progression of VD. DL-3-n-butylphthalide improved learning and cognitive impairment in VD mice by inhibiting the Nrf2/SIRT3 pathway, which reduced autophagy and apoptosis (Gao L. et al., 2022). SIRT3 also reduced neuronal apoptosis by regulating BDNF expression and synaptic plasticity (Guo et al., 2021).

Future studies may integrate blood or cerebrospinal fluid biomarkers to monitor SIRT3 activity and downstream molecular changes. Exploring the expression characteristics of SIRT3 in different brain cells such as neurons, astrocytes, and endothelial cells using single cell sequencing technology, and clarifying the feasibility of SIRT3 as a therapeutic target in VD may be also a potential research direction. Notably, cerebral small vessel disease (CSVD), recognized for its unique clinical and imaging features, progresses to vascular cognitive impairment or coexists with AD. The pathological feature of CSVD includes BBB disruption (Duering et al., 2023). Recent studies revealed that SIRT3, as a core effector molecule in the NAD^+^/SIRT3 axis, maintains BBB integrity by regulating mitochondrial metabolism, autophagy, and antioxidant defense (Zhan et al., 2025). Therefore, further research into potential pathways of SIRT3 in BBB regulation hold significant clinical value for developing targeted therapies against CSVD.

#### 4.2.2 Alzheimer’s disease

The core pathological features of AD include deposition of β-amyloid protein (Aβ), abnormal phosphorylation of microtubule associated Tau, and synaptic dysfunction (Spires-Jones and Hyman, 2014). SIRT3 regulates electron transport chain activity, stabilizes mitochondrial membrane potential, and enhances antioxidant capacity. Reduced SIRT3 expression may accelerate mitochondrial metabolic dysregulation, making neurons more susceptible to the effects of Aβ and Tau, finally accelerating neuronal apoptosis (Yin et al., 2018b). SIRT3 deficiency in GABAergic neurons exacerbates cell loss, leading to excessive hyperexcitability of neural networks and epileptiform activity. That caused the increasing mortality of AD model mice (Cheng et al., 2020). A study on APP/PS1/SIRT3^−/−^ mice revealed a significant decrease in insulin-degrading enzyme (IDE) levels in the brain compared to APP/PS1 mice. However, activation of SIRT3 by nicotinamide riboside upregulated IDE expression in normal mice, which was facilitating Aβ degradation. This study suggested SIRT3 may be a potential target for treating AD (Tyagi et al., 2022). Furthermore, SIRT3 activated the FoxO3a-SOD2 axis to mediate mitochondrial antioxidant defense system. It could reduce oxidative stress damage induced by Aβ. In AD patients, the dysfunction of this pathway may exacerbate neurodegenerative changes (Jęśko et al., 2017). In SIRT3 KO mice, the mitochondrial membrane potential of hippocampal neurons and synaptic density both decreased, and learning and memory abilities of mice were impaired. However, overexpression of SIRT3 could reduce Aβ deposition, enhance synaptic plasticity, and improve cognitive function (Yao et al., 2022). Also, SIRT3 improved neural stem cell neurogenesis via regulation of the DVL/GSK3/ISL axis, providing a theoretical basis for its clinical application (Dai et al., 2024). In chronic unpredictable mild stress mice, SIRT1 improves mitochondrial disorder and GABAergic function via SIRT1/PGC-1α/SIRT3 pathway, demonstrating their parallel contributions to neuroprotection against brain diseases (Tabassum et al., 2023).

Clinical studies confirmed that NAD^+^ precursors such as nicotinamide riboside could enhance mitochondrial metabolism by restoring SIRT3 activity, partially improving cognitive function in AD patients (Wang et al., 2021). In recent years, multiple drugs and compounds were proved to mitigate AD pathology by targeting SIRT3 (Table 3). Curcumin is a bioactive polyphenolic compound extracted from the rhizome of *Curcuma Longa L.* It could significantly improve cognitive impairment in APP_TG_ mice and alleviate neuronal metabolic dysfunction induced by Aβ_42_ though regulating the NAD^+^/NADH ratio and activating SIRT3 (Zia et al., 2021; Liu M. et al., 2021). Resveratrol, a non-flavonoid polyphenol, which not only can be extracted from the rhizome and root of *Polygonum cuspidatum Sieb. et Zucc.*, but also can be synthesized in grape leaves and skins, were widely reported to improve AD by activating the SIRT1 pathway (Surya et al., 2023). However, whether resveratrol slows down AD progression via SIRT3 activation remains to be explored. Although the important role of SIRT3 in the progression of AD has been widely recognized, targeted therapies based on SIRT3 are still in the exploratory stage. Future research should focus on discovering SIRT3 regulatory mechanisms and developing precise interventions to target its activity, offering new therapies for AD patients (Lee et al., 2018).

**TABLE 3:** SIRT3 activators that could improve Alzheimer’s disease found in nearly a decade.

Activator	Mechanism	References
Honokiol	Enhance mitochondrial SIRT3 expression and activity	Li H. et al. (2018)
Trilobatin	Modulate SIRT3/SOD2 pathway	Gao J. et al. (2022)
Salidroside	Modulate Nrf2/SIRT3 pathway	Yao et al. (2022)
Kai-Xin-San	Modulate SIRT3/NLRP3 pathway	Su et al. (2023)
ESP	Modulate Mst1/Nrf2/SIRT3 pathway	Yang et al. (2025)
PL171	Activate SIRT3 against Aβ_42_O	Li et al. (2020)
3,14,19-Triacetylandrographolide	Modulate SIRT3/FoxO3a pathway	Zhou et al. (2024)

Abbreviation: SIRT3: Sirtuin-3; SOD2: superoxide dismutase 2; Nrf2: nuclear factor erythroid 2-related factor 2; NLRP3: nucleotide-binding oligomerization domain-like receptor-related protein 3; ESP: the phenylpropanoid components of *Eleutherococcus senticosus* (Rupr. and maxim.) maxim; Mst1: mammalian sterile 20-like kinase 1; Aβ_42_O: amyloid-β_42_, oligomers; FoxO3a: forkhead box O3.

### 4.3 Movement disorders

#### 4.3.1 Parkinson’s disease

The main symptoms of PD include tremors, bradykinesia, and muscle rigidity. Degeneration of dopaminergic neurons in the substantia nigra, abnormal aggregation of α-synuclein (α-Syn), mitochondrial dysfunction, and neuroinflammation are pathological features of the disease (Trinh et al., 2023). Reduced SIRT3 expression in nigral neurons of PD patients disrupted mitochondrial function and autophagy regulation, impairing the clearance of damaged mitochondria. This resulted in α-Syn accumulation and exacerbated oxidative stress damage (Trinh et al., 2023). The abnormal expressions of SIRT3, PINK1, and TFAM in PD patients prevented neurons from mitochondria turnover and maintaining mitochondrial protein. That would aggravate oxidative phosphorylation defects and age-related oxidative stress, leading to neuronal degeneration (Chen et al., 2023). Furthermore, low SIRT3 expression cause mitophagy via PINK1/Parkin pathway, which could accelerate neuronal death, and worse PD symptoms (Gleave et al., 2017). SIRT3 knockout in 1-methyl-4-phenyl-1,2,3,6 tetrahydropyridine (MPTP)-treated PD mice exacerbated nigral neuronal degeneration and significantly reduced the expression of tyrosine hydroxylase. This process affected the synthesis of dopamine and exacerbated the movement disorders of PD (Zhang et al., 2016). Conversely, SIRT3 overexpression enhanced nigral neuronal survival, restored dopamine levels, and improved movement function (Gleave et al., 2017). It was reported that NAD^+^ metabolism regulation strategies could improve mitochondrial function and alleviate movement symptoms in PD patients (Radenkovic et al., 2020). The research on SIRT3 activators for improving PD was listed in Table 4.

**TABLE 4:** SIRT3 activators that could improve Parkinson’s disease found in nearly a decade.

Activator	Mechanism	References
s-viniferin	Modulate SIRT3/FoxO3 pathway	Zhang S. et al. (2020)
Theacrine	Direct activation of the SIRT3/SOD2 pathway	Duan et al. (2020)
Icarim	Enhanced SIRT3 activity	Zeng et al. (2019)
P7C3	Modulate Nrf2/Sirt3 pathway	Chen et al. (2024f)
Canagliflozin	Modulate PGC-1α/SIRT3 pathway	Abdelaziz et al. (2025)
Ginsenoside Rk1	Modulate SIRT3/Nrf2/HO-1 pathway	Ren et al. (2023)

Abbreviation: SIRT3: Sirtuin-3; FoxO3a: forkhead box O3; SOD2: superoxide dismutase 2; Nrf2: nuclear factor erythroid 2-related factor 2; PGC-1α: proliferator-activated receptor γ coactivator-1α; HO-1: heme oxygenase-1.

Electroacupuncture was commonly used in traditional Chinese medicine as a complementary and alternative medicine therapy for neurodegenerative diseases with minimal side effects (Zheng et al., 2021). It was shown that electroacupuncture could repair neuronal damage in PD model rats by regulating the SIRT3/NLRP3/GSDMD pathway, mitigating mitochondrial damage by clearing abnormal α-Syn accumulation in the substantia nigra (Wang et al., 2024). Additionally, electroacupuncture could activate the SIRT3/PINK1/Parkin pathway to enhance tyrosine hydroxylase expression. The aggregation of α-Syn was reduced in the MPTP-treated PD mice, and their exercise ability was improved (Zhang et al., 2024).

Notably, multiple system atrophy-parkinsonian type exhibits parkinsonian symptoms, while late-stage patients may develop cerebellar ataxia and cognitive dysfunction, with pathological features including α-Syn aggregation (Laferrière et al., 2022). However, no literature was retrieved on the relationship between SIRT3 and multiple system atrophy (MSA). Further exploration is warranted to clarify whether SIRT3 plays a role in MSA pathogenesis.

#### 4.3.2 Huntington’s disease

Huntington’s disease (HD) is a hereditary neurodegenerative disorder caused by mutations in the huntingtin protein gene. Degeneration of medium spiny neurons, synaptic dysfunction, and increased glutamate excitotoxicity are the main pathological changes, leading to choreiform movements, cognitive impairment, and psychiatric symptoms (Bates et al., 2015). Studies showed that SIRT3 expression could significantly decrease in the brains of HD patients, which correlates with overactivation of NMDA receptors mediated by NMDA receptor 2B, mitochondrial dysfunction, and impaired synaptic plasticity (Buck et al., 2017; Someya et al., 2010). Low expression of SIRT3 imbalanced excitatory synaptic, accelerating synaptic degeneration and neuronal death (Kim et al., 2019). It was reported that in models relevant to HD, SIRT3-deficient mice got worse movement dysfunction. The synaptic density of the mice decreased, while excessive activation of NMDA receptors significantly increased glutamate excitotoxicity (Cheng et al., 2016). Additionally, in HD cell models treated with the SIRT3 activator viniferin, the acetylation of SOD2 was reduced and mitochondrial function and antioxidant capacity was enhanced by activating the AMPK pathway (Fu et al., 2012). Clinical research also focuses on exploring SIRT3 as a potential therapeutic target for HD. The NAD^+^ metabolic regulation strategy was proved to improve the energy metabolism level and enhance the movement and cognitive functions of HD patients (Naia et al., 2021; Reiten et al., 2021). Furthermore, SIRT1 could active BDNF in HD striatal-like neurons, which could improve neurodegeneration and neuronal dysfunction (Duan, 2013). Thus, the synergistic roles of SIRT3 and SIRT1 in HD pathogenesis may be valuable to discover.

### 4.4 Amyotrophic lateral sclerosis

Amyotrophic lateral sclerosis (ALS) is a neurodegenerative disease affecting spinal α-motor neurons, characterized by muscle atrophy, loss of movement function, and respiratory failure. Pathological mechanisms of ALS include mitochondrial dysfunction, ROS accumulation, and protein misfolding (Suk and Rousseaux, 2020). The decrease in SIRT3 expression affected mitochondrial electron transport chain function in anterior horn motor neurons of ALS patients. This leads to insufficient energy supply to neurons, exacerbating oxidative stress damage (Hor et al., 2021). Low SIRT3 expression reduced neuronal tolerance to metabolic stress and accelerated neuronal death and muscle atrophy though AMPK/PGC-1α axis (Kuczynska et al., 2021). SIRT3 KO ALS mice showed decreased muscle strength, decreased survival rate of motor neurons, significant axonal atrophy, and myelin sheath degeneration, while the overexpression of SIRT3 attenuated neuronal damage and delayed muscle atrophy progression (Buck et al., 2017). Additionally, NAM could improve movement function and antioxidant capacity in ALS animal models (Hor et al., 2021). It was also shown that NAD^+^ precursors enhance mitochondrial bioenergetics in ALS patients and improve muscle control and movement function (Obrador et al., 2021). Although previous studies suggested that the activation of SIRT3 could reverse metabolic defects in motor neurons and alleviate symptoms, no clinical trials targeting SIRT3 were conducted in human patients to date. Future studies should advance clinical trials to explore SIRT3-targeted therapies in ALS patients.

### 4.5 Multiple sclerosis

Multiple sclerosis (MS) is a chronic demyelinating disease of the central nervous system. The neuropathology comprises inflammatory demyelination, axonal transection, and progressive neurodegeneration. Clinically, it manifests as movement disorders, sensory disturbances, visual impairment, and progressive cognitive decline (Woo et al., 2024). It was found that SIRT3 plays a critical role in maintaining energy metabolism and antioxidant defense in oligodendrocytes. Low SIRT3 expression decreased the activity of electron transport chain, promoted ROS accumulation, exacerbated neuroinflammation, and impaired remyelination capacity (Singh et al., 2018; Khodaei et al., 2019). For MS mice treated with ellagic acid, both myelin regeneration ability and movement function were improved, suggesting that SIRT3 may slow down MS progression by enhancing mitochondrial function and antioxidant capacity (Khodaei et al., 2019). Furthermore, SIRT3 regulated myelin homeostasis via the Nrf2-mediated antioxidant pathway. For MS patients, decreased expression of SIRT3 could impair myelin regeneration ability, weaken axonal protection mechanisms, and lead to disease progression (Theodosis-Nobelos and Rekka, 2022). As a potential therapeutic target for MS, intervention in its regulation strategy is expected to provide new treatment ideas for MS patients. This is beneficial for improving remyelination, slowing down disease progression, and improving patients’ quality of life.

### 4.6 Epilepsy

Epilepsy, a chronic brain disorder, arises from excessive synchronous neuronal activity in the central nervous system, accompanied by mitochondrial impairment, disrupted neurotransmitter homeostasis, and inflammatory cascades as core pathophysiological hallmarks (Milligan, 2021). A prospective observational study reported that SIRT3 levels were significantly decreased in epilepsy patients, and even lower in drug-resistant epilepsy cases (Hu et al., 2024). Research showed that blocking MciroRNA-134–5p activity preserved neuronal integrity against kainic acid neurotoxicity via SIRT3-dependent mechanisms that maintain mitochondrial homeostasis (Lin et al., 2021). Additionally, SIRT3 regulated astrocyte activation via the Notch1/NF-κB pathway, which helps alleviate the inflammatory response after epilepsy (Zhu et al., 2024). And the regulation of the NLRP3/BDNF/SIRT3 axis could reduce inflammation and oxidative stress during seizures, improving cognitive impairment caused by seizure (Fawzy et al., 2025). On the other hand, citric acid treatment in epileptic rats increased SIRT3 expression, which promoted mitochondrial autophagy and reduced hippocampal oxidative stress and apoptosis (Wu et al., 2020). It is worth noting that SIRT3 could enhance autophagy by regulating the AMPK/mTOR pathway to exert a protective effect against epilepsy induced brain damage (Chen et al. 2024a). The probability of epilepsy in patients with diabetes is significantly higher than that in normal people. Insulin could activate the SIRT1/PGC-1α/SIRT3 pathway, prolonging seizure latency, reducing seizure severity, reversing mitochondrial dysfunction, and lowering oxidative stress levels (Cheng et al., 2021). The discovery of anticonvulsant effects mediated by insulin revealed innovative strategies for health management in diabetic populations (Chou et al., 2016).

## 5 Conclusion

CNS diseases often lead to irreversible cognitive, motor, and functional impairments. Currently, these diseases generally lack effective curative therapies, making the exploration of novel neuroprotective strategies a critical direction in current research. We reviewed the core roles of SIRT3 in CNS neurons and diseases, including its critical contributions to energy metabolism, anti-oxidative stress, mitophagy, and neuroinflammation regulation. Existing studies have confirmed that the deacetylation activity of SIRT3 plays a key role in maintaining cellular homeostasis. The functions of SIRT3 may exhibit certain preferences across different types of neurons, and reduced SIRT3 activity has been observed in various CNS diseases. Therefore, it is necessary to develop SIRT3-targeted therapeutics for CNS diseases by targeting relevant pathways. However, we did not identify any clinically applicable SIRT3-targeted treatment, which may be related to the challenges in the development of BBB-penetrating drug carriers. Secondly, SIRT3 exhibits higher catalytic efficiency. Further exploring of the catalytic properties of SIRT3 is also significant. Thirdly, most current studies still focus on *in vitro* models and animal studies, where SIRT3 is activated via genetic overexpression or pharmacological activation. The clinical applicability of these approaches remains to be further validated. Notably, studies on intermittent fasting and electroacupuncture activating SIRT3 inspire us that non-pharmacological therapies may also serve as highly promising complementary alternative treatments for CNS diseases. Furthermore, since SIRT3 is expressed in major organs such as the brain, heart, and kidneys, research on SIRT3 may provide insights into the mechanisms and treatment of comorbidities such as stroke with diabetes and stroke combined with coronary heart disease. In the future, interventions targeting SIRT3, including NAD^+^ supplements, small-molecule activators, and gene modulation strategies, require systematic preclinical investigations, and their therapeutic potential in CNS diseases must be evaluated through rigorous clinical trials. In the future, interventions for SIRT3, including NAD^+^ supplements, small molecule activators, and gene regulation strategies, followed by restrictedly and scientifically designed clinical trials to evaluate their therapeutic potential in CNS.

## Glossary

CNS: central nervous system
SIRT3: Sirtuin-3
SOD2: superoxide dismutase 2
ROS: reactive oxygen species
PGC-1α: proliferator-activated receptor γ coactivator-1α
NAD+: nicotinamide adenine dinucleotide
NADPH: nicotinamide adenine dinucleotide phosphate
AD: Alzheimer’s disease
MTS: mitochondrial targeting sequence
PTM: post-translational modificationsFoxO3a forkhead box O3
Nrf2: nuclear factor erythroid 2-related factor 2
AMPK: Adenosine 5′-monophosphate (AMP)-activated protein kinase
FoxO1: forkhead box O1
KO: knockout
GABA: γ-aminobutyric acid
NMDA: N-methyl-D-aspartate
PD: Parkinson’s disease
MnSOD: superoxide dismutase
WT: wild-type
NLRP3: nucleotide-binding oligomerization domain-like receptor protein 3
ATP: adenosine triphosphate
NF-κB: nuclear factor-κB
HIF-1α: hypoxia inducible factor-1α
VEGF: vascular endothelial growth factor
STAT3: signal transducer and activator of transcription 3
Tau: protein Tau
EGR1: early growth response 1
PINK1: PTEN induced putative kinase 1
Parkin: Parkin protein
VD: vascular dementia
TFAM: transcription factor A
BDNF: brain-derived neurotrophic factor
CSVD: cerebral small vessel disease
BBB: blood-brain barrier
Aβ: β-amyloid protein
IDE: insulin-degrading enzyme
α-Syn: α-synuclein
mPTP: mitochondrial permeability transition pore
MPTP: 1-methyl-4-phenyl-1,2,3,6 tetrahydropyridine
GSDMD: Gasdermin D
MSA: multiple system atrophy
HD: Huntington’s disease
MS: Multiple sclerosis
mTOR: mammalian target of rapamycin
